# The function of Sphingosine-1-phosphate receptor 2 (S1PR2) in maintaining intestinal barrier and inducing ulcerative colitis

**DOI:** 10.1080/21655979.2022.2076500

**Published:** 2022-06-15

**Authors:** Tanzhou Chen, Kaier Gu, Ruoyang Lin, Yang Liu, Yunfeng Shan

**Affiliations:** a The Department of Gastroenterology and Hepatology, The First Affiliated Hospital of Wenzhou Medical University, Wenzhou, People’s Republic of China; b Surgery, The First Affiliated Hospital of Wenzhou Medical UniversityThe Department of Hepato-Pancreato-Biliary, Wenzhou, People’s Republic of China

**Keywords:** S1PR2, IECs barrier, ulcerative colitis, permeability

## Abstract

Sphingosine-1-phosphate receptor 2 (S1PR2) was highly expressed in intestinal epithelial cells (IECs) and facilitated the proliferation of IECs. However, the specific function of S1PR2 in intestinal diseases, such as ulcerative colitis (UC), remains unclear. Accordingly, the current study set out to investigate the function of S1PR2 in maintaining intestinal barrier and inducing UC. S1PR2-overexpressed and knockdown Caco-2 cells were established to explore the function of S1PR2 on the permeability of IECs barrier. The UC-like mouse model in which UC is induced by dextran sulfate sodium (DSS) was established and utilized to investigate the role for S1PR2. The results showed that S1PR2 functioned as a maintainer of IECs permeability and a pathogenic factor for UC. A series of in vitro and in vivo experiments were conducted, and it was found that S1PR2 played an important role in intestinal epithelial cell proliferation and maintenance of intestinal epithelial cell barrier, possibly by the regulation on the expression level of SphK2, HDAC1, HDAC2, and ERK1/2 signaling pathway. The expression of S1PR2 was upregulated in UC mice and the colonic pathological damage in UC mice could be alleviated by the inhibition of S1PR2. Collectively, these results suggest that S1PR2 functions as a maintainer of IECs permeability and a pathogenic factor for UC. The research suggests S1PR2 may be an effective target for developing therapeutic strategies against UC.

**Abbreviations:** S1PR2, Sphingosine-1-phosphate receptor 2; UC, ulcerative colitis; IECs, intestinal epithelial cells; DSS, dextran sulfate sodium; IBD, inflammation bowel disease; CD, Crohn’s disease; S1P, sphingosin-1-phosphate; SphK, sphingosine kinase; HIECs, human IECs; siRNA, small interfering RNA; CCK-8, cell counting kit-8; TEER, transepithelial electrical resistance; TEM, transmission electron microscope; RT-PCR, real-time reverse transcriptase polymerase-chain reaction; ELISA, enzyme-linked immunosorbent assay; HE, hematoxylin and eosin.

## GRAPHICAL ABSTRACT

**Figure:**
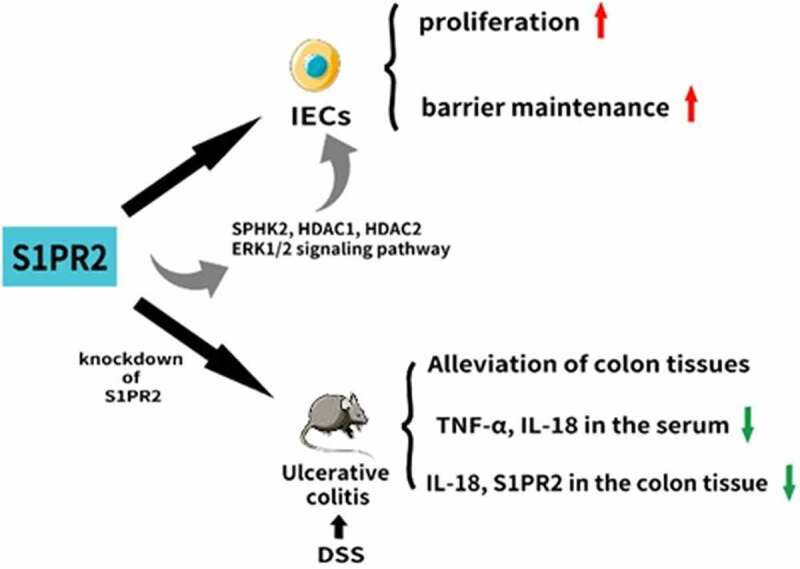

## Highlights

- S1PR2 promote cell proliferation and increase cellular permeability in intestinal epithelial cells.
- The knockdown of S1PR2 ameliorates the pathological injury caused by dextran sodium sulfate.
- S1PR2 exerts an important role possibly mediated by the regulation on the expression level of SphK2, HDAC1, HDAC2, and ERK1/2 signaling pathway.
- S1PR2 might be an effective target for developing therapeutic strategies against UC.

## 1. Introduction

Ulcerative colitis (UC) is a nonspecific chronic inflammatory bowel disease, which is characterized by continuous diffuse inflammation, damage to the intestinal mucosal barrier, and excessive immune, accompanied by clinical symptoms, such as abdominal pain, diarrhea, rectal bleeding, bloody stools, and weight loss [1]. The mortality rate of UC in Western countries is relatively high, and the Asian region has recently shown an upward trend year by year [2,3]. At present, the treatments of UC are mainly resolution of inflammation and promotion of intestinal mucosal wound repair [4,5]. Therefore, it is extremely important to explore novel targets on anti-inflammation and intestinal mucosal repair.

Intestinal mucosal barrier is an important barrier against the invasion of external environment, including epithelial barrier, immune barrier, and microbial barrier, among which intestinal epithelial barrier is regarded as the most important barrier and the basis for the selective permeability of intestinal mucosa. The intestinal epithelial barrier comprises intestinal epithelial cells (IECs) and the tight junctions between them, which regulates the transport of water and solutes across the epithelium [6]. Several junctions are reported to connect IECs, including tight junctions, adherent junctions, gap junctions, and desmosomes, among which tight junctions are essential for the maintaining of intestinal mucosal barrier [7]. Damages on tight junctions in the intestinal epithelium is reported to be involved in the pathogenesis of multiple types of diseases. Therefore, it is of great significance to maintain intact tight junctions between IECs to protect intestinal barrier function and prevent bacterial migration and toxic macromolecules from entering the body. Intestinal mucosal barrier dysfunction due to cellular permeability changes is a key factor in the pathogenesis of UC.

Many inflammatory cytokines, such as members of the IL-1 family, play an important role in intestinal homeostasis and inflammation [8]. IL-18 has emerged as an indispensable factor in the regulation of host-microbial homeostasis and has been postulated to be a critical inducer for the development of UC [9]. It is reported that the level of IL-18 is closely associated with the severity of IBD and can be utilized as the biomarker for IBD [10]. However, recently, several researches reveal that mice with IL-18 or IL-18 R knockout are more sensitive, rather than resistant, to dextran sulfate sodium (DSS)-induced enteritis and inflammatory bowel cancer [11]. Collectively, IL-18 plays a dual role in the development of enteritis, promoting the progression of enteritis as an inflammatory factor, yet inducing tissue repair to prevent enteritis.

Sphingosine-1-phosphate (S1P) is a bioactive sphingolipid metabolite on the membrane, which is involved in many important physiological and pathophysiological processes including cell growth, cell apoptosis, cell proliferation, and migration [12]. S1P is synthesized via the phosphorylation of sphingosine by sphingosine kinase (SphKs). There are two isoforms of SphKs called SphK1 and SphK2. SphK1 has been described to be present mainly in the cytosol while SphK2 is mainly located in the nucleus [13]. S1P is reported to exert its biological functions by directly acting as an intracellular signaling molecule or extracellularly activating five G-protein-coupled receptors (S1PRs) extracellularly [14,15]. S1PRs are expressed differently across the tissues [15]. All five S1PRs are expressed in the human intestine, however, with different expression levels. It is reported that S1PR1 protects IL-10^−/−^ mice from colitis by reducing epithelial cell apoptosis and improving intestinal barrier function [16]. In addition, S1P is reported to delay the apoptosis in IECs through the Akt-dependent pathway [17]. These researches indicate that S1P and its receptors exert protective effects on the intestinal mucosal barrier function by promoting the proliferation of IECs and enhancing intestinal epithelial cell barrier function. However, the role of S1PR2 in proliferation of IECs and intestinal epithelial barrier function remains unclear and seems worth investigating further.

Our previous study revealed that the proliferation and migration of IECs could be promoted by S1P, S1PR2 was highly expressed in IECs and S1P-mediated cell proliferation and migration is mainly mediated by S1PR2 [18]. Inhibition of S1PR2 activity with S1PR2 inhibitor JTE-013 prevented S1P-induced cell proliferation, accompanied by downregulation of IL-18. S1PR2 plays an important role in intestinal epithelial cell proliferation and IL-18 release, maintaining the intestinal epithelial cell barrier. Additionally, activation of the S1PR2 results in activation of ERK1/2 signaling pathway, which further activates SphK2. S1P produced in the nucleus by SphK2 inhibits HDAC1/2 activity and increases histone acetylations, and then promote gene expression. Guided by these findings, we hypothesized that S1PR2 might alleviate UC by repairing the intestinal epithelial cell barrier via promoting the proliferation of IECs, inducing the release of IL-18, and regulating on the expression level of SphK2, HDAC1, HDAC2, and ERK1/2 signaling pathway.

## 2. Materials and methods

### 2.1. Cell culture and treatment

Human colon cancer cell line Caco-2 cells and human IECs (HIECs) were purchased from ATCC (California, USA). Caco-2 cells were cultured in MEM medium (KGM41500-500, Keygen biotech, Nanjing, China) supplemented with the mixture of penicillin and streptomycin (S G0200, olarbio, Beijing, China) and 10% FBS (10,099–141, GibcoTM, Grand Island, USA). HIECs were cultured in 1640 medium (1065–018, Keygen biotech, Nanjing, China) supplemented with the mixture of penicillin and streptomycin (G0200, Solarbio, Beijing, China) and 10% FBS (10,099–141, GibcoTM, Grand Island, USA). Cell supernatants and cells were collected and analyzed for the presence of cytokine proteins and gene expression. In some experiments, TNF-α (0332078JQ27, Novoprotein Biotech Co., Suzhou, China), the S1PR2-specific inhibitor (JTE-013, 49,951, MCE), the S1PR2-specific agonist (CYM5520, C5098, and APEXBIO), and S1P (S9666, sigma) were added to the culture immediately before the start of the culture, or the siRNA targeting S1PR2 vector and plasmid mediated S1PR2 overexpression vector were added simultaneously, or scramble RNA and empty vector were added simultaneously. The siRNA targeting S1PR2 vector, plasmid mediated S1PR2 overexpression vector, scramble RNA and empty vector were all purchased from Zhonghong Boyuan (Shenzhen) Biotechnology Co., Ltd. and performed according to the manufacturer’s instructions.

### 2.2. Cell transfection

Small interfering RNA (siRNA) against S1PR2, scramble siRNA, pcDNA3.1-S1PR2 overexpression vector and pcDNA3.1 empty vector were all purchased from Zhonghong Boyuan (Shenzhen) Biotechnology Co., Ltd. and performed according to the manufacturer’s instructions. Transfection was performed in cells with siRNA targeting S1PR2, S1PR2 scramble siRNA, S1PR2 overexpression vector or empty vector. The knockdown of S1PR2 was achieved with siRNA targeting S1PR2 (siRNA-1, siRNA-2, and siRNA-3), and S1PR2 scramble siRNA was used as a negative control (siRNA NC). The overexpression of S1PR2 was achieved with pcDNA3.1-S1PR2 vector (S1PR2), and the pcDNA3.1 empty vector was used as a negative control (NC). Cells were transfected with vectors using Lipofectamine 3000 (Thermo Fisher Science, Waltham, MA, USA. The targeting sequence for each S1PR2 siRNA is listed in Supplemental Table S1.

### 2.3. Cell counting kit-8 (CCK-8) assay

Cells were mixed with 10 µL CCK-8 solution (Cat. No: KGA317, Keygen biotech, Nanjing, China) and incubated at 37°C for 2 h, and the absorbance at 450 nm was analyzed via a microplate reader (J&H technology Co., Ltd, Jiangsu, China) to determine the cell viability.

### 2.4. The detection of fluorescein permeability

The detection of fluorescein permeability, an important indicator of cell transport and barrier function, could measure the changes in transport capacity. Treated cells were seeded on the 96-well plate at the density of 2 × 10^5^ cells/well and incubated for 3–4 days to form a monolayer. Cells were then carefully washed with PBS for 3 times and incubated at 37°C for 30 min. 80 μg/mL fluorescent yellow (MKCJ3736, Sigma, Missouri, USA) was added to the top of Transwell and the liquid on the substrate side was collected after incubation for 1 h. The absorbance (excitation wavelength 427 nm and emission wavelength 536 nm) was measured under a fluorescence spectrophotometer (Thermo Fisher, MA, USA). The concentration of fluorescent yellow was calculated according to the standard curve and the result was expressed as the clearance rate of fluorescent yellow. Cl[nL /(h•cm2)] = Lab/([LY]a × S). Cl: clearance rate, Lab: the fluorescein permeability from the top side to the base side, [LY] a: the basic amount of fluorescence, S: the area of the Transwell.

### 2.5. Transepithelial electrical resistance (TEER) measurement

TEER was an important indicator reflecting the integrity of barrier and cell membrane permeability. Transepithelial permeability was assessed by the value of TEER. Treated cells were implanted in a 96-well plate at the density of 2 × 10^5^ cells/well and incubated for 3–4 days to form a monolayer. Compounds JTE-013 (10 μM) and CYM5520 (10 μM) were added in advance and incubated for 1 h. After adding TNF-α (100 ng/mL) to be incubated for 24 h, the resistance was measured and analyzed by an electrical resistance meter (Thermo Fisher, MA, USA).

### 2.6. Transmission electron microscope (TEM)

Cells were collected and washed using PBS buffer, followed by fixed in 4.0% glutaraldehyde in PBS overnight. Subsequently, cells were embedded in epoxy resin and ultrathin sections (50–70 nm) were collected on copper grids. After counterstaining with aqueous uranyl acetate for 1 h, phosphotungstic acid for 1 h, and Reynolds’ lead citrate for 20 min successively, followed by examined on a TEM (JEOL, Tokyo, Japan).

### 2.7. Establishment of a DSS-induced UC mouse model

6-8-week male C57BL/6 mice were purchased from Hunan Slyke Jingda Experimental Animal Co., LTD. They were housed in temperature- and humidity-controlled rooms kept on a 12 h light/dark cycle and provided unrestricted amounts of food and water; the bedding was changed twice a week. After 1 week of adaptive feeding, animals were randomly divided into six groups: (1) control group; (2) DSS group; (3) DSS+ JTE-013 group; (4) DSS+ CYM5520 group; (5) DSS+ NC group; (6) DSS+ si-S1PR2 group.

For the establishment of DSS induced UC model, mice were fed with drinking water containing 2% DSS (H07D11B13349, Shanghai YuanYe Biotechnology Co., Ltd., Shanghai, China) for one week, followed by being fed with the normal drinking water for another one week. In the control group, mice were fed with normal drinking water for 2 weeks. In the DSS+ JTE-013 group, UC mice were simultaneously dosed with 10 mg/kg/day JTE-013 orally for one week. In the DSS+ CYM5520 group, UC mice were simultaneously dosed with 10 mg/kg/day CYM5520 orally for one week. In the control group, UC mice were simultaneously dosed with an equal volume of normal saline orally for one week. In the DSS group, UC mice were simultaneously dosed with an equal volume of DMSO solvent orally for one week. UC mice in the DSS+ NC group and DSS+ si-S1PR2 group were injected in situ with lentiviral containing si-NC and si-S1PR2 4 days prior to the finish of DSS feeding, respectively. On the 14^th^ day post DSS feeding, animals were dosed orally with 600 mg/kg fluorescein isothiocyanate-dextran, followed by being fasted and deprived with water for 4 hours prior to sacrificing. Samples were collected for the subsequent experiments. All animal experiments were reviewed and approved by the Laboratory Animal Ethical Committee of laboratory Animal Center, Whenzhou Medical University (Approval number: wydw2018-0084).

### 2.8. Real-time reverse transcriptase polymerase chain reaction (RT-PCR)

The TRIZOL© reagent (CW0580S, CWBIO, Beijing, China) and Ultrapure RNA extraction kit (CW0581M, CWBIO, Beijing, China) was utilized for the extraction of total RNAs from colon tissues, which were further reverse-transcribed to cDNA using the TaqMan miRNA reverse transcription kit (Invitrogen. California, USA). The RT-PCR was conducted using HiScript II Q RT SuperMix (R223-01, Vazyme, Nanjing, China) with the 2× SYBR Green PCR Master Mix (A4004M, Lifeint, Xiamen, China), the conditions of which were 94°C for 5 min., 30 cycles of 94°C for 30 sec, and 58–61°C for 30 sec, followed by 72°C for 2 min. β-actin was used for the normalization of relative expression of target genes, which was calculated using the 2^−ΔΔCt^ method. The sequences for the primers were shown in Table 1.

**Table 1.** The sequences of the primers

Name	Sequences (5’-3’)
IL-18 F(h)	ATAATGCACCCCGGACCATA
IL-18 R(h)	ATGTCCTGGGACACTTCTCT
β-actin F(h)	TGGCACCCAGCACAATGAA
β-actin R(h)	CTAAGTCATAGTCCGCCTAGAAGCA
S1PR2 F(m)	ATGGGCGGCTTATACTCAGAGF
S1PR2 R(m)	GCGCAGCACAAGATGATGAT
IL-18 F(m)	AGACTCTTGCGTCAACTTCAA
IL-18 R(m)	GAGGGTAGACATTTTACTATCCTTC
β-actin F (m)	TGGCACCCAGCACAATGAA
β-actin R(m)	CATACCCAAGAAGGAAGGCT

### 2.9. Western blotting assay

Following extracting total proteins from tissues using the lysis buffer, a BCA kit (Cwbiotech, Beijing, China) was used to quantify the isolated proteins and approximately 40 μg proteins were loaded and separated by the 12% SDS PAGE, followed by being transferred to the PVDF membrane (Millipore, Massachusetts, USA). The membrane was then incubated with 5% BSA (Solarbio, Beijing, China) followed by incubation with the primary antibody against p-ERK1/2 (1:500, AF1015, Affintiy, Melbourne, Australian), S1PR2 (1:500, 21,180-1-AP, Proteintech, Wuhan, China), SphK2 S1PR2 (1:500, 17,096-1-AP, Proteintech, Wuhan, China), HDAC1 (1:500, 10,197-1-AP, Proteintech, Wuhan, China), HDAC2 (1:500, 12,922-3-AP, Proteintech, Wuhan, China), and GAPDH (1:2000, Zsbio, Beijing, China). Subsequently, the membrane was incubated with the secondary antibody (1:2000, Zsbio, Beijing, China) at room temperature for 1.5 h. Lastly, the bands were visualized by ECL solution and the relative expression level of target proteins was quantified by the Image J software.

### 2.10. Enzyme-linked immunosorbent assay (ELISA)

ELISA was used to measure the production of TNF-α and IL-18 in colon tissues. Briefly, testing samples and standards were implanted in 96-well plates, followed by being added with the conjugate reagents to be incubated for 1.5 h. After adding the TMB solution to each well, samples were incubated at 37°C for 15 min. Lastly, the stop solution was added to terminate the reaction, followed by measuring the absorbance at 450 nm using the microplate reader (Mindray, Shenzhen, China).

### 2.11. Hematoxylin and eosin (HE) staining

After collecting the colon tissues from each animal and washing, tissues were dehydrated with 70%, 80%, and 90% ethanol solution successively, followed by being incubated with equal quality of ethanol and xylene for 15 min. Subsequently, the samples were incubated with equal quality of xylene for another 15 min, followed by repeat incubation until the tissues looked transparent. Then, the tissues were embedded in paraffin, sectioned, and stained with HE staining, followed by randomly selecting the images from five fields at 100× magnification were captured and all phases of follicles and corpora lutea were count by using an inverted microscope (Olympus, Tokyo, Japan). The severity of tissue damage was evaluated with histopathological score (H-score) based on four parameters as indicated in Supplemental Table S2.

### 2.12. Statistical analysis

The GraphPad software was used to analyze achieved data, which were expressed as mean ± SD. The Student’s t-test was used to analyze data between two groups and data among more than three groups were analyzed using the one-way ANOVA method. P < 0.05 was considered a significant difference in the present study.

## 3. Results

The study aimed to investigate the function of S1PR2 in maintaining intestinal barrier and inducing UC. A series of in vitro and in vivo experiments were conducted, and it was found that S1PR2 played an important role in intestinal epithelial cell proliferation and maintenance of intestinal epithelial cell barrier, possibly by the regulation on the expression level of SphK2, HDAC1, HDAC2, and ERK1/2 signaling pathway. The expression of S1PR2 was upregulated in UC mice and the colonic pathological damage in UC mice could be alleviated by the inhibition of S1PR2. Our data revealed that S1PR2 might be an effective target for developing therapeutic strategies against UC.

### 3.1. The effects of S1PR2 on epithelial barrier in Caco2 cells

**Figure 1.**
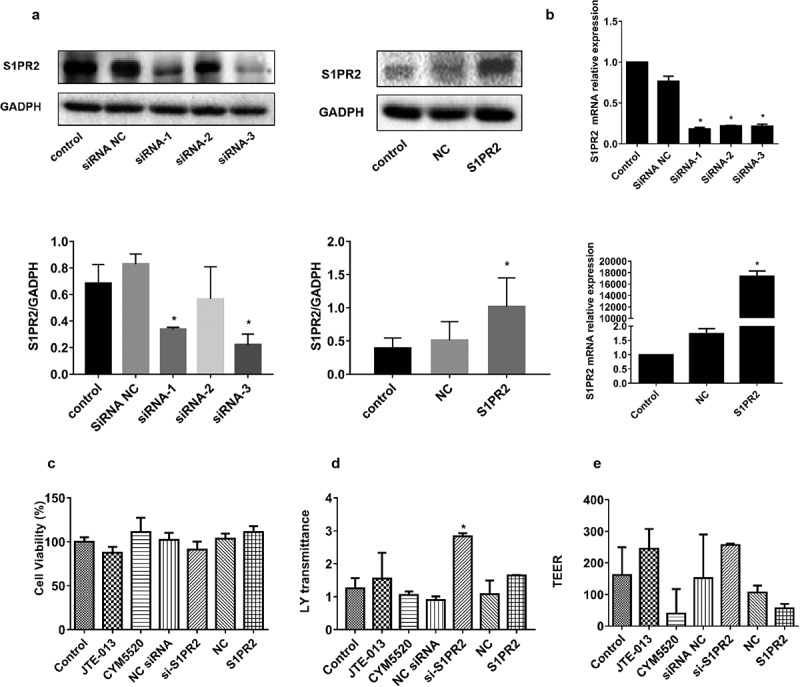
The impacts of S1PR2 on the epithelial barrier established by Caco2 cells. A. The transfection efficacy of siRNAs and pcDNA3.1-S1PR2 was identified by the Western blotting assay (*p < 0.05 vs. siRNA NC or NC). B. The transfection efficacy of siRNAs and pcDNA3.1-S1PR2 was identified by the RT-PCR assay (*p < 0.05 vs. siRNA NC or NC). C. The cell viability was measured by the CCK-8 assay. D. The impact on material transportation was evaluated by the fluorescein permeability (*p < 0.05 vs. control). E. The resistance values were determined by the electrical resistance meter.

To overexpress and inhibit S1PR2 in Caco2 cells, the vectors containing pcDNA3.1-S1PR2 and S1PR2 siRNAs were established, respectively. Caco2 cells were transfected with S1PR2 siRNA (siRNA-1, siRNA-2, and siRNA-3), S1PR2 scramble siRNA (siRNA NC), pcDNA3.1-S1PR2 (S1PR2), or pcDNA3.1 empty vector (NC) for 48 h. The expression of S1PR2 was examined using Western blotting analysis and the expression of mRNA was examined using RT-PCR. As shown in Figure 1(a), S1PR2 was significantly downregulated in Caco2 cells transfected with siRNA-1 and siRNA-3 (*p < 0.05 vs. siRNA NC), while S1PR2 was significantly upregulated in Caco2 cells transfected with pcDNA3.1-S1PR2 (*p < 0.05 vs. NC). Similar results have been achieved in the results of RT-PCR (Figure 1(b)). These data suggested that transfection with pcDNA3.1-S1PR2 vector resulted in a significant increase of S1PR2 levels compared with empty pcDNA3.1 vector, and transfection with siRNA targeting S1PR2 resulted in a significant decrease of S1PR2 levels compared with S1PR2 scramble siRNA (*p < 0.05 vs. siRNA NC or NC). Based on these results, siRNA-1 was selected for subsequent experiments.

Caco2 cells established the epithelial barrier model after 21-day incubation. Caco2 cells were transfected with S1PR2 siRNA (siRNA-1, siRNA-2, and siRNA-3), S1PR2 scramble siRNA (siRNA NC), pcDNA3.1-S1PR2 (S1PR2), or pcDNA3.1 empty vector (NC) for 48 h or treated with JTE-013 (10 μM) or CYM5520 (10 μM) for 1 h, respectively. Subsequently, 100 ng/mL TNF-α was introduced to be incubated for 24 h. The cell viability was measured by the CCK-8 assay. The impact on material transportation was evaluated by the fluorescein permeability. The intestinal epithelial cell barrier function was evaluated by measuring the TEER. As shown in Figure 1(c), compared to control group, slightly declined cell viability was observed in the JTE-013 and si-S1PR2 group, while slightly increased cell viability was observed in the CYM5520 and S1PR2 group. Additionally, compared to the JTE-013 group, the cell viability was elevated in the CYM5520 group; compared to si-S1PR2 group, the cell viability was elevated in the S1PR2 group. These data suggested that S1PR2 could promote cell proliferation in human colon cancer cell line Caco-2 cells.

The detection of fluorescein permeability, an important indicator of cell transport and barrier function, could measure the changes in transport capacity. As shown in Figure 1(d), compared to control group, the fluorescein permeability was slightly elevated in the JTE-013 group and slightly reduced in the CYM5520 group. Additionally, compared to siRNA NC the fluorescein permeability was significantly elevated in the si-S1PR2 group (*p < 0.05 vs. siRNA NC).

TEER was an important indicator reflecting the integrity of barrier and cell membrane permeability. Transepithelial permeability was assessed by the value of TEER. As shown in Figure 1(e), compared to control group, slightly promoted resistance value was observed in the JTE-013 and si-S1PR2 group, while slightly declined resistance value was observed in the CYM5520 and S1PR2 group.

These data suggested that S1PR2 could increase cellular permeability in human colon cancer cell line Caco-2 cells.

### 3.2. The impacts of S1PR2 on the morphology changes in tight junctions of epithelial barrier in Caco2 cells

**Figure 2.**
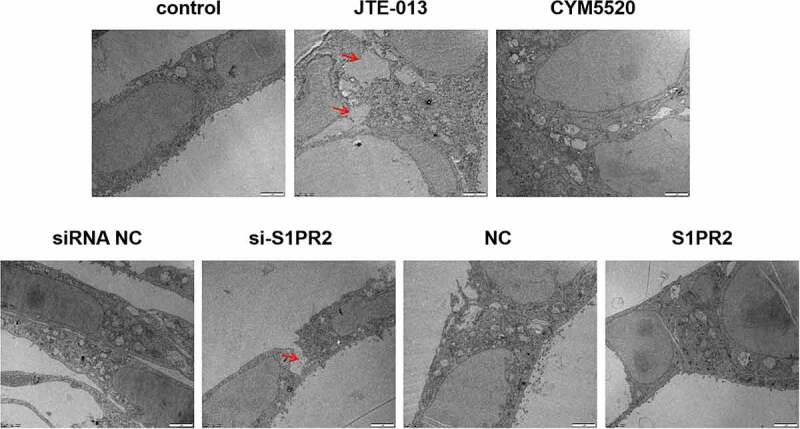
The morphological changes of tight junctions in the epithelial barrier established by Caco2 cells was visualized by TEM (2 μm). Arrow (→) indicates area of the structure of tight junction was significantly disrupted.

As shown in Figure 2, clear tight junction was observed in the control, CYM5520, and siRNA NC group. However, the structure of tight junction was significantly disrupted in the JTE-013 and si-S1PR2 group, while higher degree of tightness was observed in the S1PR2 group. These data suggested that S1PR2 was critical for the structure of tight junction in the epithelial barrier.

### 3.3. The effects of S1PR2 on the proliferation of S1P stimulated Caco2 cells and HIECs

**Figure 3.**
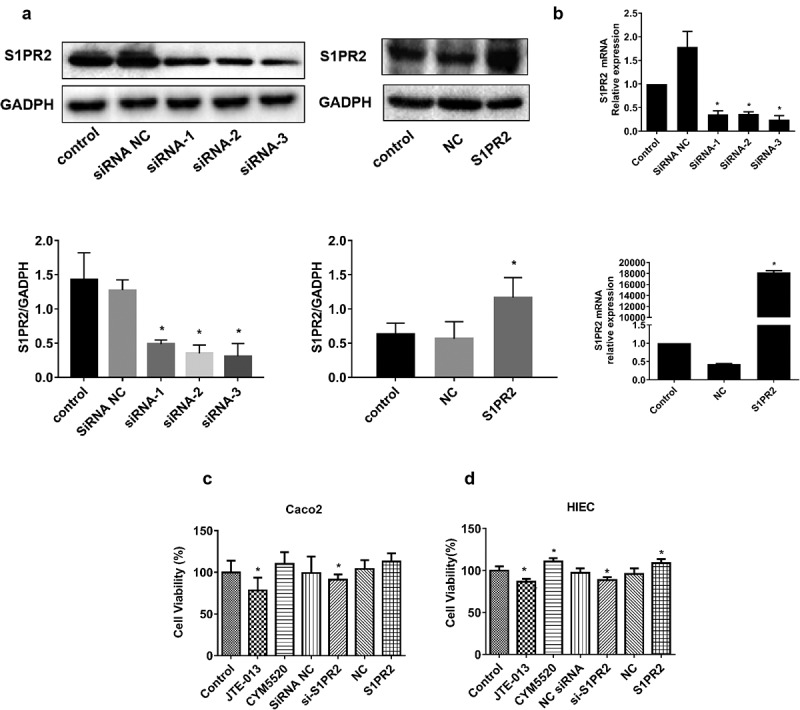
The impacts of S1PR2 on the proliferation of S1P-stimulated Caco2 cells or HIECs. A. The transfection efficacy of siRNAs and pcDNA3.1-S1PR2 was identified by the Western blotting assay (*p < 0.05 vs. siRNA NC or NC). B. The transfection efficacy of siRNAs and pcDNA3.1-S1PR2 was identified by the RT-PCR assay (*p < 0.05 vs. siRNA NC or NC). C. The cell viability in Caco2 cells was measured by the CCK-8 assay (*p < 0.05 vs. control). D. The cell viability in HIECs was measured by the CCK-8 assay (*p < 0.05 vs. control).

To overexpress and inhibit S1PR2 in HIECs, the vectors containing pcDNA3.1-S1PR2 and S1PR2 siRNAs were established, respectively. HIECs were transfected with S1PR2 siRNA (siRNA-1, siRNA-2, and siRNA-3), S1PR2 scramble siRNA (siRNA NC), pcDNA3.1-S1PR2 (S1PR2), or pcDNA3.1 empty vector (NC) for 48 h. The expression of S1PR2 was examined using Western blotting analysis and the expression of mRNA was examined using RT-PCR. As shown in Figure 3(a), S1PR2 was significantly downregulated in HIECs transfected with all three siRNAs (*p < 0.05 vs. siRNA NC), while S1PR2 was significantly upregulated in HIECs transfected with pcDNA3.1-S1PR2 (*p < 0.05 vs. NC). Similar results have been achieved in the results of RT-PCR (Figure 3(b)). Based on these results, siRNA-1 was selected for subsequent experiments.

Caco2 cells or HIECs established the epithelial barrier model after 21-day incubation. Cells were transfected with S1PR2 siRNA (siRNA-1, siRNA-2, and siRNA-3), S1PR2 scramble siRNA (siRNA NC), pcDNA3.1-S1PR2 (S1PR2), or pcDNA3.1 empty vector (NC) for 48 h or treated with JTE-013 (10 μM) or CYM5520 (10 μM) for 1 h, respectively. Subsequently, 100 ng/mL S1P was introduced to be incubated for 24 h. The cell viability was measured by the CCK-8 assay. As shown in Figure 3(c), in Caco-2 cells, compared to control group, the cell viability was significantly repressed in the JTE-013 and si-S1PR2 group (*p < 0.05 vs. control), while the cell viability was slightly elevated in the CYM5520 and S1PR2 group. As shown in Figure 3(d), in HIECs, compared to control group, the cell viability was significantly repressed in the JTE-013 and si-S1PR2 group, while the cell viability was significantly elevated in the CYM5520 and S1PR2 group (*p < 0.05 vs. control). These data suggested that S1PR2 could promote cell proliferation in IECs, whether in human colon cancer cell line Caco-2 cells or in HIECs.

### 3.4. The impact of S1PR2 on the expression level of S1PR2, SphK2, HDAC1/2, and p-ERK1/2 in the epithelial barrier

**Figure 4.**
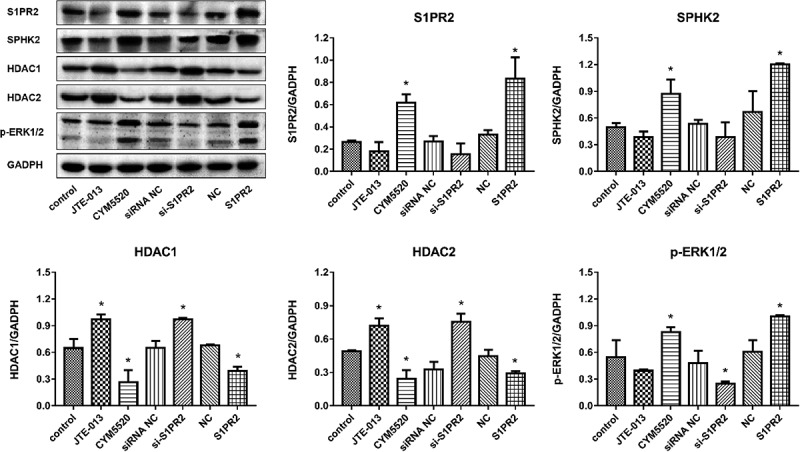
The impacts of S1PR2 on the expression level of S1PR2, SphK2, HDAC1/2, and p-ERK1/2 in S1P-stimulated Caco2 cells were evaluated by the Western blotting assay (*p < 0.05 vs. control).

**Figure 5.**
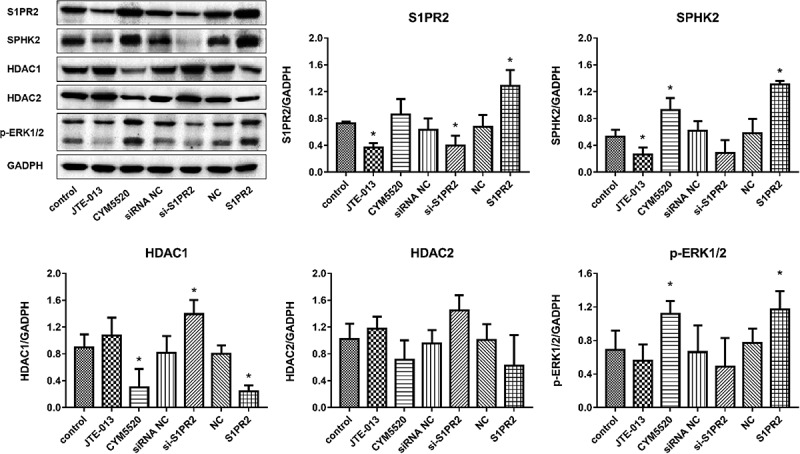
The impacts of S1PR2 on the expression level of S1PR2, SphK2, HDAC1/2, and p-ERK1/2 in S1P-stimulated HIECs were evaluated by the Western blotting assay (*p < 0.05 vs. control).

In Caco-2 cells, as shown in Figure 4, compared to control group, S1PR2 and SphK2 were found slightly downregulated in the JTE-013 and si-S1PR2 group and dramatically upregulated in the CYM5520 and S1PR2 group. The expression level of HDAC1/2 was significantly elevated in the JTE-013 and si-S1PR2 group and was greatly reduced in the CYM5520 and S1PR2 group. Additionally, compared to control group, p-ERK1/2 was slightly downregulated in the JTE-013 and significantly downregulated in the si-S1PR2 group, while the expression level of p-ERK1/2 was dramatically promoted in the CYM5520 and S1PR2 group (*p < 0.05 vs. control). In HIECs, as shown in Figure 5, compared to control group, S1PR2 was found significantly downregulated in the JTE-013 and si-S1PR2 group, slightly upregulated in the CYM5520 group, and significantly upregulated in the S1PR2 group. Compared to control group, SphK2 was significantly downregulated in the JTE-013 group, slightly downregulated in the si-S1PR2 group, and significantly upregulated in the CYM5520 and S1PR2 group. Additionally, compared to control group, the expression level of HDAC1/2 was slightly elevated in the JTE-013 and si-S1PR2 group and was slightly reduced in the CYM5520 and S1PR2 group. Lastly, compared to control group, p-ERK1/2 was slightly downregulated in the JTE-013 and the si-S1PR2 group and dramatically upregulated in the CYM5520 and S1PR2 group (*p < 0.05 vs. control). These data suggested that increased the upregulation of S1PR2 leads to activation of the ERK1/2 pathway, which further increases SphK2 activity and reduces HDAC1/2 expression, thereby maintaining intestinal barrier.

### 3.5. The pathological changes in the colon tissues of UC mice

**Figure 6.**
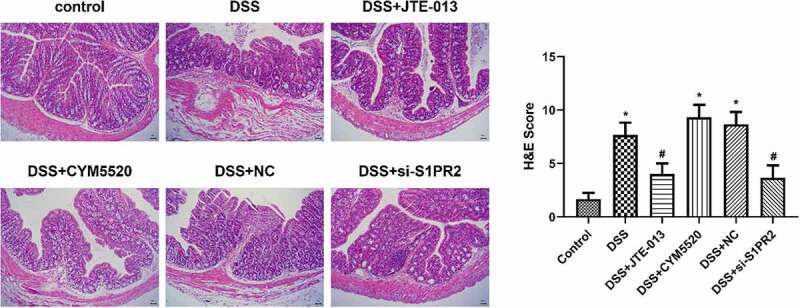
The knockdown of S1PR2 reduces DSS-induced colonic mucosal damage. (Left) HE staining was used to assess the degree of intestinal mucosal injury of mice in each group; (Right) Histological score of colons were used to assess the degree of intestinal mucosal injury of mice in each group (*p < 0.05 vs. control, #p < 0.05 vs. DSS).

As shown in Figure 6, no obvious pathological changes were observed and the colon tissue structure was intact in the control group, with rarely congestion, ulcers, and necrosis. The colonic epithelium was intact with clear structure and the intestinal gland was arranged orderly. However, in the DSS group, a large number of necrotic and exfoliated colon epithelial cells, glandular damage, irregular and disorderedly arranged structures, secretions in the cavity, and infiltration of a large number of inflammatory cells were observed. Compared to the model group, the pathological state of colon tissues in the DSS+ JTE-013 group was alleviated greatly, with rare infiltration of inflammatory cells and orderly arranged epithelial cells. The colonic pathology in DSS+CYM5520 and DSS+ NC group was basically consistent with the DSS group. However, in the DSS+ si-S1PR2 group, less hyperemia, mild damaged epithelial cells with intact structures, and a small amount of infiltrated inflammatory cells were observed. Consistent with the histological findings, compared to control group, the histological scores of UC mice was significantly elevated in the DSS group (*p < 0.05 vs. control). Additionally, compared to the DSS group, the histological scores of UC mice was significantly declined in the JTE-013 and si-S1PR2 group (#p < 0.05 vs. DSS). These data suggested that the inhibition of S1PR2 could alleviate the colonic pathological damage in UC mice.

### 3.6. The changes of TNF-α and IL-18 concentration in the serum of UC mice

**Figure 7.**
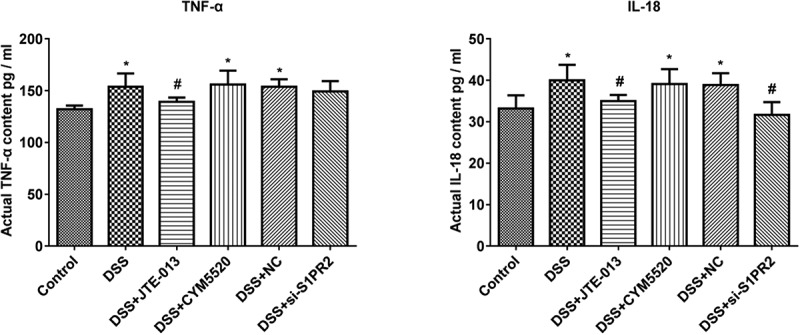
The impacts of S1PR2 on the changes of TNF-α and IL-18 concentration in the serum were evaluated the ELISA. Bar charts represent the concentration of TNF-α (Left) and IL-18 (Right) in the serum in each group (*p < 0.05 vs. control, #p < 0.05 vs. DSS).

As shown in Figure 7, compared to control group, the levels of TNF-α and IL-18 were significantly higher in the DSS group (*p < 0.05 vs. control). Compared to the DSS group, the levels of TNF-α and IL-18 were greatly repressed in the DSS+ JTE-013 and DSS+ si-S1PR2 group (#p < 0.05 vs. DSS). These data suggested that DSS led to the substantial expression of TNF-α and IL-18 in the serum. Additionally, the inhibition of S1PR2 could reduce the high levels of TNF-α and IL-18 induced in UC mice.

### 3.7. The changes of S1PR2 and IL-18 protein level in the colon tissue of UC mice

**Figure 8.**
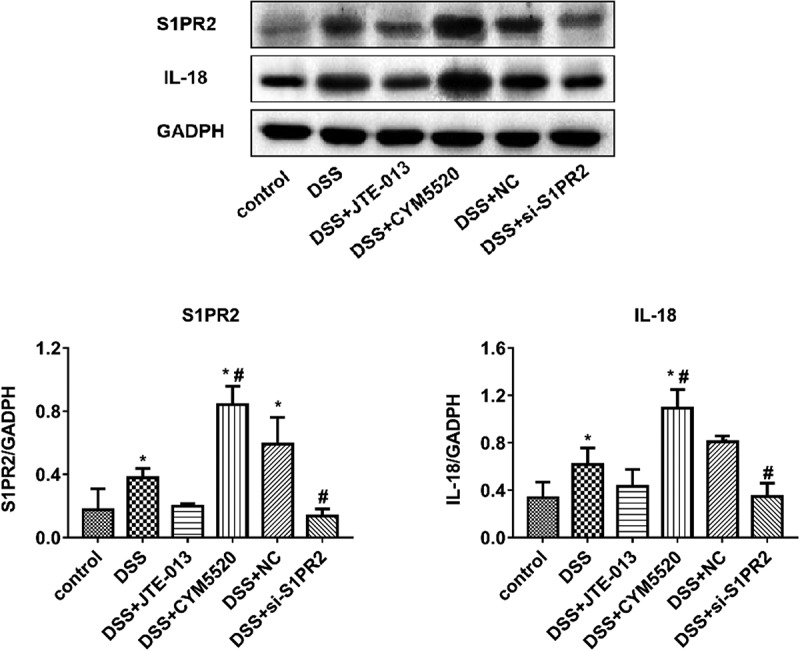
The impacts of S1PR2 on the changes of S1PR2 and IL-18 protein level in colon tissue were evaluated by the Western blotting assay. Bar charts represent the concentration of S1PR2 (Left) and IL-18 (Right) in colon tissue in each group (*p < 0.05 vs. control, #p < 0.05 vs. DSS).

As shown in Figure 8, compared to control group, the levels of S1PR2 and IL-18 were dramatically upregulated in the DSS group (*p < 0.05 vs. control). Compared to the DSS group, the levels of S1PR2 and IL-18 were greatly downregulated in the DSS+ si-S1PR2 group (#p < 0.05 vs. DSS), which were slightly downregulated in the DSS+ JTE-013 group. Additionally, compared to the DSS group, the levels of S1PR2 and IL-18 had no significant changes in the DSS+ NC group, which were greatly elevated in the DSS+CYM5520 group (#p < 0.05 vs. DSS). These data suggested that DSS upregulated the expression of S1PR2 in the colon tissue. Additionally, the inhibition of S1PR2 could inhibit the production of IL-18, and the molecular mechanism of DSS damage ameliorated by the inhibition of S1PR2 may be due to the inhibition of IL-18 expression. However, the regulation of IL-18-mediated signaling pathway regulated by S1PR2 remains poorly understood.

## 4. Discussion

UC is a recurrent disease induced by inflammation in the gastrointestinal system. Recently, the morbidity of UC increases annually, which triggers the development of novel treatment strategies [19–21]. However, despite great efforts, the treatment for UC remains a clinical challenge. In the present study, in vitro experiments revealed that S1PR2 played an important role in promoting the proliferation of IECs and maintaining the integrity of the intestinal epithelial barrier, which might be associated with the regulation on the expression level of SphK2, HDAC1, HDAC2, and the ERK1/2 signaling pathway. In vivo experiments revealed that S1PR2 expression was upregulated in UC mice. The inhibition of S1PR2 could reduce the high levels of IL-18 and colonic mucosal damage in UC mice, suggesting that S1PR2 might be a potential target for the treatment of UC.

S1P is a biologically active sphingolipid metabolite synthesized by SphKs through sphingosine phosphorylation and is involved in many important physiological and pathophysiological processes, such as cell growth, cell apoptosis, cell proliferation, and migration [12]. There are two isoforms of SphKs called SphK1 and SphK2. SphK1 has been described to be present mainly in the cytosol while SphK2 is mainly located in the nucleus [13]. SphK1 is known to translocate the plasma membrane driven by many agonists. For example, cytokines induce extracellular signal-regulated kinase (ERK)-dependent phosphorylation of SphK1 in a Ca^2+^/Camodulin-dependent manner [22]. Export of SphK2 to the cytoplasm relies on PMA-induced protein kinase D-mediated phosphorylation of SphK2[23]. S1P regulates signaling through a variety of receptors, including five different S1PRs. S1PRs are expressed differently across the tissues [15]. S1PR1/2/3 are widely expressed in a variety of tissues, while S1PR4 is restrictedly expressed in lymphoid and hematopoietic tissues and S1PR5 is mainly expressed in the central nervous system, indicating each S1PR plays overlapping but also unique physiological and pathophysiological roles. All five S1PRs are expressed in the human intestine, however, with different expression levels. Researches indicate that S1P and its receptors exert protective effects on the intestinal mucosal barrier function by promoting the proliferation of IECs and enhancing intestinal epithelial cell barrier function [16,17]. S1PR2 has been confirmed to be positively correlated with the occurrence and progression of inflammation in various diseases [24–26]. Additionally, research has shown that genes of S1P synthesis (SphK1, SphK2), degradation (SGPL1), and signaling (S1PR1, S1PR2, and S1PR4) were significantly upregulated in colon biopsies of IBD patients with moderate/severe symptoms compared with controls or patients in remission [27].

Our previous study revealed that the proliferation and migration of IECs could be promoted by S1P, S1PR2 was highly expressed in IECs and S1P-mediated cell proliferation and migration is mainly mediated by S1PR2 [18]. Inhibition of S1PR2 activity with S1PR2 inhibitor JTE-013 prevented S1P-induced cell proliferation, accompanied by downregulation of IL-18. S1PR2 plays an important role in intestinal epithelial cell proliferation and IL-18 release, maintaining the intestinal epithelial cell barrier. Guided by these findings, the present study aimed to investigate the function of S1PR2 in maintaining intestinal barrier and inducing UC.

Our in vitro experiments showed that in the epithelial barrier established by S1PR2-knockdown Caco2 cells, declined cell viability, increased fluorescein permeability and resistance value, and destruction of tight junctions were observed. These results indicate that S1PR2 could promote cell proliferation and increase cellular permeability in Caco-2 cells. Additionally, S1PR2 was critical for the structure of tight junction in the epithelial barrier. In the epithelial barrier established by S1P treated Caco2 cells and HIECs, downregulation of S1PR2, SphK2, and p-ERK1/2 was observed by the knockdown of S1PR2. These results indicate that increased the upregulation of S1PR2 leads to activation of the ERK1/2 pathway, which further increases SphK2 activity and reduces HDAC1/2 expression, thereby maintaining intestinal barrier.

HDAC1 and HDAC2 are direct targets of S1P. The coinhibitory complex of cyclin-dependent kinase inhibitor P21 can directly bind to SphK2 and S1P in the nuclear promoter region, thereby inhibiting HDAC function and enhancing histone H3 acetylation, thereby promoting the transcription process effect [28]. In sgpl1-deficient mouse embryonic fibroblasts, HDAC activity is reduced due to the accumulation of nuclear S1P [29]. In addition, HDAC inhibitor treatment has a significant inhibitory effect on inflammation and tumor development [30]. In our study, inhibition of S1PR2 significantly increased the expression levels of HDAC1 and HDAC2 in Caco2 cells and HIECs, indicating that S1PR2 has potential anti-inflammatory properties. Further studies for these speculations are needed.

Our in vivo experiments showed that S1PR2 and IL-18 in the colon tissue were significantly upregulated in UC mice. The expression level of S1PR2 and IL-18 was repressed in the colon tissue of S1PR2-knockdown UC mice. The colonic pathological damage in UC mice was alleviated by the knockdown of S1PR2, accompanied by the decreased concentration of TNF-α and IL-18 in the serum of mice. The molecular mechanism of DSS damage ameliorated by the inhibition of S1PR2 may be due to the inhibition of IL-18 expression. However, the regulation of IL-18-mediated signaling pathway regulated by S1PR2 remains poorly understood.

## 5. Conclusion

Our results suggested that S1PR2 played an important role in intestinal epithelial cell proliferation and maintenance of intestinal epithelial cell barrier, possibly by the regulation on the expression level of SphK2, HDAC1, HDAC2, and ERK1/2 signaling pathway. The expression of S1PR2 was upregulated in UC mice and the colonic pathological damage in UC mice could be alleviated by the knockdown of S1PR2. Our data revealed that S1PR2 might be an effective target for developing therapeutic strategies against UC.

## Supplementary Material

Supplemental Material
