# Supplementary material for: The function of Sphingosine-1-phosphate receptor 2 (S1PR2) in maintaining intestinal barrier and inducing ulcerative colitis
Source: Bioengineered. 2022 Jun 15;13(5):13703–17. doi: 10.1080/21655979.2022.2076500 (PMC9276026; doi:10.1080/21655979.2022.2076500)
Supplement: Supplemental Material [file KBIE_A_2076500_SM7043.zip › 2076500/Supplemental_Table_1.docx]

Table 1 The sequences of the primers

| **Name** | **Sequences (5’-3’)** |
| --- | --- |
| IL-18 F(h) | ATAATGCACCCCGGACCATA |
| IL-18 R(h) | ATGTCCTGGGACACTTCTCT |
| β-actin F(h) | TGGCACCCAGCACAATGAA |
| β-actin R(h) | CTAAGTCATAGTCCGCCTAGAAGCA |
| S1PR2 F(m) | ATGGGCGGCTTATACTCAGAGF |
| S1PR2 R(m) | GCGCAGCACAAGATGATGAT |
| IL-18 F(m) | AGACTCTTGCGTCAACTTCAA |
| IL-18 R(m) | GAGGGTAGACATTTTACTATCCTTC |
| β-actin F (m) | TGGCACCCAGCACAATGAA |
| β-actin R(m) | CATACCCAAGAAGGAAGGCT |
