# Supplementary material for: The function of Sphingosine-1-phosphate receptor 2 (S1PR2) in maintaining intestinal barrier and inducing ulcerative colitis
Source: Bioengineered. 2022 Jun 15;13(5):13703–17. doi: 10.1080/21655979.2022.2076500 (PMC9276026; doi:10.1080/21655979.2022.2076500)
Supplement: Supplemental Material [file KBIE_A_2076500_SM7043.zip › 2076500/Supplemental_Table_2.docx]

Supplemental Table 2 Histological score of colons

| **Score** | **Inflammation severity** | **Mucosa damage** | **Crypt damage** | **Lesion range (%)** |
| --- | --- | --- | --- | --- |
| 0 | none | none | none | 0 |
| 1 | slight | mucosal layer | basal 1/3 damaged | 1-25 |
| 2 | moderate | submucosal layer | basal 2/3 damaged | 26-50 |
| 3 | severe | muscle layer | only surface epithelium intact | 51-75 |
| 4 | very severe | transmural | entire crypt and epithelium lost | 76-100 |
